# Biomimetic remineralization of acid etched enamel using agarose hydrogel model

**DOI:** 10.12688/f1000research.16050.1

**Published:** 2018-09-17

**Authors:** Sara El Moshy, Marwa M.S. Abbass, Amal M. El-Motayam

**Affiliations:** 1 Oral Biology Department, Faculty of Dentistry, Cairo University, Cairo, 11553, Egypt

**Keywords:** Remineralization, agarose, enamel, microhardness, surface roughness.

## Abstract

**Background**: Minimally invasive dentistry aims to prevent progression of caries and treats non-cavitated lesions through non-invasive approaches to preserve the integrity of tooth structure. The aim of this research was to investigate the possible biomimetic effect of agarose hydrogel in remineralizing a human demineralized enamel model.

**Methods**: Mandibular third molars were distributed into three groups (G1, G2 and G3) according to the follow up time (2, 4 and 6 days respectively). Caries like lesion was prepared by applying 37% phosphoric acid gel for 1 minute and then remineralization was performed through applying agarose hydrogel on the demineralized surfaces. The specimens were placed in phosphate solution at 37˚C for 2, 4 & 6 days. Scanning electron microscope (SEM), surface microhardness (SMH) and surface roughness analysis (SR) were performed to assess the regenerated tissue.

**Results**: SEM revealed mineral depositions on the demineralized enamel surface that increased in density by time resulting in a relatively smooth surface in G3. SR and SMH analysis revealed significant differences between the remineralized enamel surfaces of different groups (p< 0.00001) with the highest SR in G1 and the highest SMH in G3.

**Conclusions**: Agarose hydrogel application is a promising approach to treat early carious lesion. Further studies are needed to clarify the stability of agarose hydrogels in clinical application.

## Introduction

Biomimetic remineralization is a non-invasive therapeutic approach that has received great attention in the last decades. It aims to restore the dental tissues to its normal biological function and esthetics ^1^. Although several studies have proposed different methods to remineralize enamel lesions, their clinical applications are limited because they require difficult application conditions ^2– 5^. Agarose is a natural biocompatible polysaccharide that has been proposed as a matrix for crystal formation ^6– 9^. Therefore, the purpose of this study was to investigate the possible biomimetic effect of agarose hydrogel in remineralizing a human demineralized enamel model.

## Methods

### Specimens preparation

The experiment was done according to the recommendations and approval of the Ethics Committee of the Faculty of Dentistry, Cairo University for working on extracted human teeth (Approval no.18766). Mandibular third molars were collected after being surgically extracted due to impaction with patients' written consents. The roots of 47 tooth were removed using diamond disk (Komet, Rock Hill, USA, K6974) in low speed under water cooling. The crowns were divided mesio-distally and each half was embedded in self-cured acrylic resin (Acrostone Co. Cairo, Egypt, 01CCP50) exposing the uncovered enamel surface. Specimens were examined under stereomicroscope (Leica S8 APO, Leica Microsystems, Switzerland) and specimens with defects (erosions, cracks, visible stains, hypo-calcification) were excluded. Specimens were distributed into three groups (n = 31/ group), according to follow up time ( Table 1). Specimens were demineralized using 37% phosphoric acid gel (Super Etch, SDI Limited, Australia, 8100040) for 1 min and rinsed with de-ionized water for 60 seconds.

**Table 1.** Specimens grouping and intervention.

Group	Demineralization	Remineralization	Time of application	Follow up
**G1** **(n = 31)**	37% phosphoric acid gel for 1 min.	Agarose hydrogel 2mm thickness	48 hours	2 days
**G2** **(n = 31)**			96 hours, hydrogel changed every 48 hours.	4 days
**G 3** **(n = 31)**			144 hours, hydrogel changed every 48 hours.	6 days

G1 (2 days), G2 (4 days), G3 (6 days).

### Remineralization

Agarose (Vivantis, USA, PC0701) hydrogel and phosphate solution were prepared as previously mentioned by Cao *et al*., ^7^. Agarose hydrogel was applied on the specimen using acrylic template of 2mm thickness to adjust the thickness of the applied hydrogel. After gelation of the applied hydrogels each specimen was placed into a container filled with 20 mL of phosphate solution and placed in an incubator at 37°C. The phosphate solution and the hydrogel were changed every 24 and 48 h respectively.

### Scanning electron microscope (SEM) examination

Thirteen specimens from each group were mounted on the SEM plate with electro-conductor glue (Electron Microscopy Sciences, PA, USA, 12660) to examine their surfaces. The used SEM Model was Quanta FEG 250 (Field Emission Gun) with accelerating voltage 30 K.V.

### Surface microhardness (SMH) analysis

SMH of 9 specimens from each group was measured using microhardness tester with Vickers diamond indenter in different areas of the specimens (Vickers diamond, 100 g, 5 s, HMV 2; Shimadzu Corporation, Tokyo, Japan). SMH was measured at baseline, after demineralization and after remineralization.

### Surface roughness (SR) analysis

SR of 9 specimens from each group was measured using digital microscope equipped with a built-in camera (Digital Microscope U500X, Guangdong, China). The microscope is connected to IBM compatible computer. WSxM software (Version 5 develop 4.1, Nanotec, Electronica, SL) was used to analyze the photos and to create a 3D image of the specimen surface. The average SR was estimated using WSxM software and expressed in µm. SR was measured at baseline, after demineralization and after remineralization.

### Statistical analysis

The mean SMH values and the mean SR values were statistically analyzed. One-way ANOVA followed by Tukey's post hoc test were performed to compare remineralizing potential at different time intervals (2,4,6 days). Furthermore, the same tests were used to compare enamel surfaces within the same group. The significant level was set at 0.05. Statistical analysis was performed with SPSS 18.0 for Windows (Statistical Package for Scientific Studies, SPSS, Inc., Chicago, IL, USA).

## Results

### SEM examination

**Figure 1.**
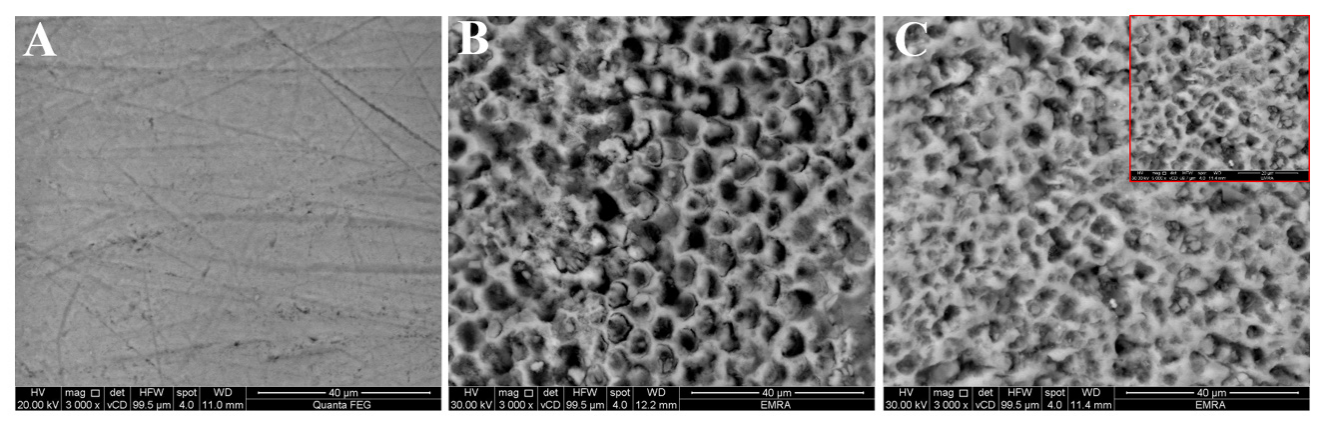
Scanning electron microscope (SEM) images for G1; sound enamel at baseline ( **A**), demineralized enamel surface ( **B**), remineralized enamel surface ( **C**).

**Figure 2.**
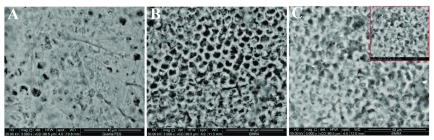
Scanning electron microscope (SEM) images for G2; sound enamel at baseline ( **A**), demineralized enamel surface ( **B**), remineralized enamel surface ( **C**).

**Figure 3.**
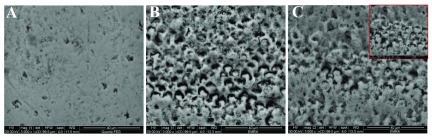
Scanning electron microscope (SEM) images for G3; sound enamel at baseline ( **A**), demineralized enamel surface ( **B**), remineralized enamel surface ( **C**).

Sound enamel has a smooth surface with some pits and scratches ( Figure 1A, Figure 2A & Figure 3A). After acid etching different etching patterns were seen, most commonly type I and type II with scattered areas of type III ( Figure 1B, Figure 2B & Figure 3B). After remineralization, G1 revealed partial occlusion of some rod cores with clearly thickened interprismatic substance ( Figure 1C) while in G2 prismatic enamel configurations became hidden by mineral depositions ( Figure 2C). G3 revealed a relatively smooth surface with less clearly seen rod ends. Some rods’ peripheries showed complete remineralization while others were still empty ( Figure 3C).

### SMH analysis

The mean SMH values of enamel at different intervals (2,4,6 days) are presented in Table 2. In G1, significant differences were revealed between baseline, demineralized and remineralized enamel (p<0.05) with the highest SMH at baseline. While in G2 and G3, there was a significant difference between the baseline and the demineralized enamel (p<0.05), however there wasn’t a significant difference between baseline and remineralized enamel. Furthermore, there were significant differences among the remineralized enamel surfaces of different groups (p<0.05) with the highest SMH at G3.

**Table 2.** Analysis of Surface microhardness (SMH) (Kgf/mm ^2^).

	SMH-B	SMH-D	SMH-R	P-value
**G1 (2D)**	254.377±24.73 ^a^	171.138±15.23 ^c^	196.864±9.74 ^b,C^	< 0.00001*
**G2 (4D)**	251±35.88 ^a^	171.84±32.42 ^b^	218.485±14.76 ^a,B^	0.000028 ^*^
**G3 (6D)**	256.842±24 ^a^	175±8.98 ^b^	242.433±14.36 ^a;A^	<0 .00001 ^*^
**P-value**	0.911069	0.918935	< 0.00001 ^*^	

Baseline (B), after demineralization (D), after remineralization (R). Different upper and lower-case superscript letters indicate significant difference between tested groups at P<0.05. Lower case superscript letters are used for comparison within the same row and upper case letters are used for comparison within each column.

### SR analysis

**Figure 4.**
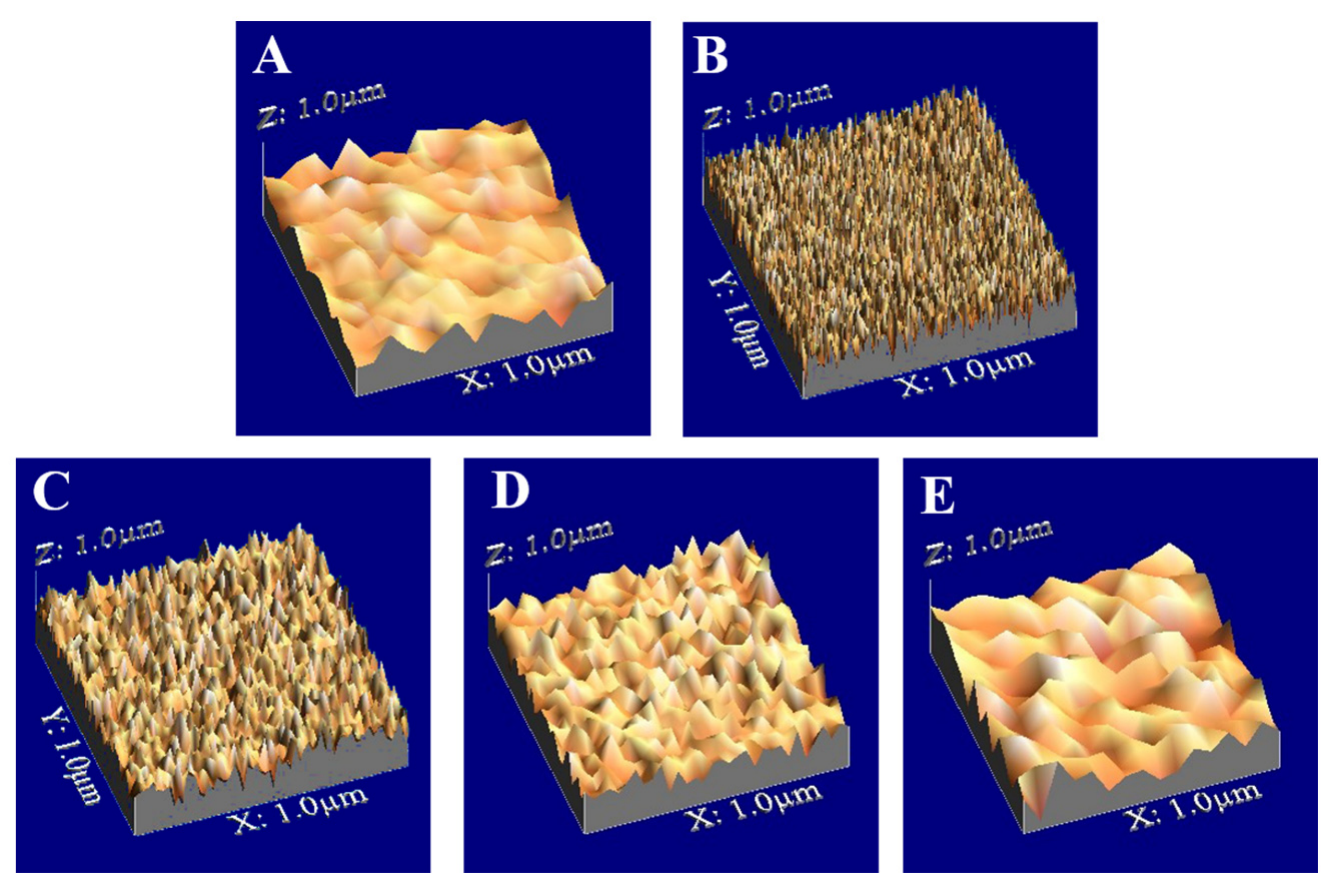
Representative Surface roughness (SR) images of enamel specimens; baseline **A**, demineralized enamel **B**, remineralized enamel surfaces **C**, **D**, **E** (G1, G2, G3 respectively).

The mean SR values of enamel at different intervals (2,4,6 days) are presented in Table 3. In G1, there were significant differences between baseline, demineralized and remineralized enamel (p<0.05) with the highest SR at the demineralized enamel. While in G2 and G3, there was a significant difference between the baseline and the demineralized enamel (p<0.05), however there wasn’t a significant difference between baseline and the remineralized enamel. Furthermore, there were significant differences among the remineralized enamel surfaces of different groups (p<0.05) with the highest SR in G1. The differences in SR at baseline, demineralized enamel and after remineralization in different groups were obvious when inspecting the 3D images in Figure 4.

**Table 3.** Analysis of Surface roughness (SR) (µm).

	SR-B	SR-D	SR-R	P-value
**G1 (2D)**	0.253±0.0009 ^c^	0.274±0.0025 ^a^	0.2663±0.002 ^b,A^	< 0.00001 ^*^
**G2 (4D)**	0.256±0.0096 ^b^	0.275±0.0026 ^a^	0.258±0.003 ^b,B^	< 0.00001 ^*^
**G3 (6D)**	0.254±0.00027 ^b^	0.275±0.003 ^a^	0.255±0.003 ^b,C^	< 0.00001 ^*^
**P-value**	0.376997	0.508623	< 0.00001 ^*^	

Baseline (B), after demineralization (D), after remineralization (R). Different upper and lower-case superscript letters indicate significant difference between tested groups at P<0.05. Lower case superscript letters are used for comparison within the same row and upper case letters are used for comparison within each column.

Raw surface microhardness (SMH) and surface roughness (SR) Click here for additional data file. Copyright: © 2018 El Moshy S et al. 2018 Data associated with the article are available under the terms of the Creative Commons Zero "No rights reserved" data waiver (CC0 1.0 Public domain dedication).

Raw scanning electron microscope (SEM) images Click here for additional data file. Copyright: © 2018 El Moshy S et al. 2018 Data associated with the article are available under the terms of the Creative Commons Zero "No rights reserved" data waiver (CC0 1.0 Public domain dedication).

## Discussion

Biomimetic synthesis of enamel like apatite structures under a physiological condition is an alternative restorative pathway ^10^. Acid etching technique was used to mimic early enamel lesions because of the simplicity and reproducibility of this technique ^11^. SEM results of the present study are in agreement with previous studies ^6– 9^. Agarose hydrogel acted as enamel organic matrix to control the size and form of the formed hydroxyapatite crystals through the interaction between hydroxyl group of agarose and calcium. In addition, it acts as a mineral reservoir for continuing remineralization ^7^. The SR analysis results confirmed the SEM results, as the SR values were gradually decreased between different groups which revealed a smoother enamel surface. SMH results are in accordance with previous studies ^7, 9^. In the current work, the lower SMH than sound enamel could be attributed to incomplete compaction of formed crystals on enamel surface ^12^.

## Conclusions

Agarose hydrogel model have a remineralizing potential to treat early carious lesion. Further studies are required to clarify the stability of agarose hydrogels in clinical application.

## Data availability

The data referenced by this article are under copyright with the following copyright statement: Copyright: © 2018 El Moshy S et al.

Data associated with the article are available under the terms of the Creative Commons Zero "No rights reserved" data waiver (CC0 1.0 Public domain dedication). 

Dataset 1: Raw surface microhardness (SMH) and surface roughness (SR) 10.5256/f1000research.16050.d217398 ^13^

Dataset 2: Raw scanning electron microscope (SEM) images 10.5256/f1000research.16050.d217399 ^14^
