# Supplementary material for: Biomimetic remineralization of acid etched enamel using agarose hydrogel model
Source: F1000Res. 2018 Sep 17;7:1476. [Version 1] doi: 10.12688/f1000research.16050.1 (PMC6178904; doi:10.12688/f1000research.16050.1)
Supplement: Peer review history

# Peer review: Referee response for version 1

**DOI:** 10.5256/f1000research.17529.r39249

**Published:** 2018-10-17

**Authors:** Mahmoud M. Bakr

**Affiliations:** 1 School of Dentistry and Oral Health, Griffith University, Nathan, Qld, Australia

The study by El Moshy *et al.* is well designed and investigates an important topic with potential clinical applications.

 However, there are a few issues that need to be addressed to improve the quality of the manuscript:

1. Introduction and discussion sections are extremely short and do not cite enough literature on the topic.
2. Discussion section besides being short is basically a repetition of some results without discussing technical aspects of the study and comparing it to previous similar studies.
3. The quality of some of the images could be improved.
4. The statistical analysis is not a true representation of the results and could be inaccurate. The effect of time is neglected in this case. Ideally a two way ANOVA should be used to illustrate the interaction between time and treatment. If no interaction was observed then the single main effects of time and/or treatment could be reported then. Using the post hoc analysis is not the best practice as it neglects the effect of time and will most likely lead to false-positive results. 
5. Some images for illustration of the techniques used in the materials and methods section would be helpful.

I have read this submission. I believe that I have an appropriate level of expertise to confirm that it is of an acceptable scientific standard.

# Peer review: Referee response for version 1

**DOI:** 10.5256/f1000research.17529.r39250

**Published:** 2018-10-15

**Authors:** Mahmoud M. Al-Ankily

**Affiliations:** 1 Oral Biology Department, Faculty of Dentistry, British University in Egypt, Cairo, Egypt

This report by El Moshy et al. examines biomimetic remineralization of acid etched enamel using agarose hydrogel model. The authors' inclusion of Agarose hydrogel model has a remineralizing potential to treat early carious lesion. The study, although it is small, adds knowledge to the existing literature.

 Minor comments would help to improve the impact of this paper:

**Methods**

 Specimens preparation: It is better to use premolars extracted for orthodontic treatment, the surface of enamel is usually intact and standard in enamel rods orientation, crystallization and size rather than mandibular third molars with the anatomical varieties, surgical instrumentation and surface irregulars.

 Remineralization: (Agarose hydrogel and phosphate solution were prepared as previously mentioned) there is no previously mentioned information about hydrogels and Phosphate Solution Preparation.

 Surface roughness (SR) analysis: The average SR was expressed in 1 μm only. how can you be sure that you measure SR at baseline, after demineralization and after remineralization of the same 1 μm every time. It is recommended to use another type of surface roughness analysis with wider surface area at least 25 μm like AFM.

    

**Results**

 SEM examination: Figures x3000 is not good enough to show baseline, after demineralization and after remineralization changes also x5000 is not so clear please do not minimize them to save the magnification benefits. It is recommended to use 20000 to 50000 to show the crystals, regenerated mineralized tissue and crystal orientation.

**Discussion**

 Needs more details about mechanism of growth of the enamel crystals and the mechanical properties of the regeneration tissue.

I have read this submission. I believe that I have an appropriate level of expertise to confirm that it is of an acceptable scientific standard.

# Peer review: Referee response for version 1

**DOI:** 10.5256/f1000research.17529.r39209

**Published:** 2018-10-15

**Authors:** Mohamed Shamel

**Affiliations:** 1 Oral Biology Department, Faculty of Dentistry, Modern Sciences and Arts University, Cairo, Egypt

The current study performed by El Moshy el al, is an interesting one that aims to investigate the possible biomimetic effect of agarose hydrogel in remineralizing a human demineralized enamel model.

 Overall, the study is well constructed and presented with the results efficiently supporting the discussion and conclusions.

 However some minor points might be helpful in adding to the study:

- SEM images used were croped from the original images which caused some blurriness in the images.
- A more detailed statistical analysis needs to be performed on the relatively large amount of data obtained.
- The discussion section needs to be more detailed as it is too short in comparison to the results obtained.

I have read this submission. I believe that I have an appropriate level of expertise to confirm that it is of an acceptable scientific standard.

# Peer review: Referee response for version 1

**DOI:** 10.5256/f1000research.17529.r38419

**Published:** 2018-10-05

**Authors:** Nehad Samir Taha

**Affiliations:** 1 Oral Biology Department, Faculty of Oral and Dental Medicine, Misr International University (MIU), Cairo, Egypt

Excellent work:

 1. The study design is appropriate to the work done.

 2. Replication of this study could be applicable in the future based on the study results.

 3. The statistical analysis is appropriate to the study.

 4. In my opinion, the study results efficiently support the conclusions drawn. 

 But, I had some questions about:

 1. The acrylic templates: how to use it, and whether it is standard in size.

 2. No references for the etching pattern.

 3. Some pictures are hazy and with no arrows.

 Further comments:

 1. In the Methodology: I would prefer if you could show us in one picture the differences between a defected specimen that you excluded and one that you choose.

 2. In the Results:

- In Figure 1: no arrows to demonstrate the different types of acid etching.
- In Figure 1C: no arrows to show the closed rod cores.
- In Figure 2C: no arrows to show the areas of mineral deposition.
- No reference to the acid etching classification.
- Finally, I preferred to see a figure plate comparing the 3 states together to be easy to compare (i.e.: a plate of Figures 1A, 2A and 3A, a plate of Figures 1B, 2B and 3B, and a plate of Figures 1C, 2C and 3C).

 

 3. The discussion is too short to clarify the findings of the study.

I have read this submission. I believe that I have an appropriate level of expertise to confirm that it is of an acceptable scientific standard.

# Peer review: Referee response for version 1

**DOI:** 10.5256/f1000research.17529.r38418

**Published:** 2018-09-20

**Authors:** Raneem Farouk Obeid, Radwa Taher el sharkawy

**Affiliations:** 1 Department of Oral Biology, Future University in Egypt, New Cairo, Egypt; 2 Faculty of Dentistry, Future University in Egypt, New Cairo, Egypt

Good work, but I have some comments to clarify my confusion:

1. In the Methodology: the acrylic template - why and how to use? And do you standardize the 2mm in this template?
2. In the Results:
   
  - In Figure 2 you mention acid etch type? Where is the reference of this classification and arrows to show the different type?
  - Picture C is hazy, please change it.
  - Figure 3: please put arrows to show us rod complete and empty one in Picture C.

We have read this submission. We believe that we have an appropriate level of expertise to confirm that it is of an acceptable scientific standard.
